# Twig-like middle cerebral artery: a case report and literature review

**DOI:** 10.3389/fcvm.2025.1530241

**Published:** 2025-03-11

**Authors:** Kai-Meng Wang, Yan-Zhao Xie, Guo-Dong Xu, Wentao Yao, He-Bo Wang

**Affiliations:** 1 The Neurology Department of Hebei General Hospital, Shijiazhuang, Hebei, China; 2 The Neurointervention Department of Hebei General Hospital, Shijiazhuang, Hebei, China

**Keywords:** twig-like middle cerebral artery, cerebrovascular angiography, neuroimaging, pathogenesis, differential diagnosis, clinical manifestation

## Abstract

**Background:**

Twig-like middle cerebral artery (T-MCA) is a rare cerebrovascular malformation with an incidence of approximately 0.11% to 1.17%. It is challenging to distinguish T-MCA from Moyamoya Angiopathy (MMA), particularly from unilateral MMA.

**Case presentation:**

A middle-aged female patient was admitted to our hospital for diagnosis and treatment due to intermittent dizziness lasting for two months. Neurological examination upon admission revealed no significant abnormalities.Whole-brain angiography showed clear blood flow in the left common carotid artery and internal carotid artery; however, no main trunk of the distal left middle cerebral artery M1 segment was visualized, and a network of small arteries supplying blood was observed. High-resolution intracranial arterial plaque imaging revealed no visualization of the LM1 main trunk on plain scan or enhanced high-resolution black-blood sequence; the proximal segment showed significant narrowing with circumferential thickening of the vessel wall. The patient was ultimately diagnosed with left-sided T-MCA.

**Conclusions:**

Asymptomatic T-MCA is challenging to identify clinically, making it essential to perform DSA or high-resolution MRI for suspected cases promptly. Regular follow-up is also necessary to confirm the final diagnosis in these patients.

## Introduction

Twig-like middle cerebral artery (T-MCA) is a rare cerebrovascular cerebrovascular malformation with an incidence of approximately 0.11% to 1.17%. Most T-MCA patients are found to have abnormalities in the middle cerebral artery due to headaches, dizziness, or during routine examinations through magnetic resonance angiography (MRA) or computed tomography angiography (CTA), and the diagnosis is further confirmed by transcranial cerebral angiography (1). The most important differential diagnosis for T-MCA is moyamoya angiopathy (MMA). Due to the similarity in vascular structures, it is challenging to distinguish T-MCA from MMA, particularly from unilateral MMA. T-MCA patients may be asymptomatic in the early stages, but serious complications, such as aneurysms and vascular ruptures, can occur. Therefore, early clinical recognition of T-MCA is crucial to avoid complications and reduce inappropriate interventions. This article reports a case of a patient who presented with simple dizziness and was diagnosed with T-MCA through digital subtraction angiography (DSA), aiming to raise clinical awareness and attention to this condition.

## Case report

**Figure 1:**
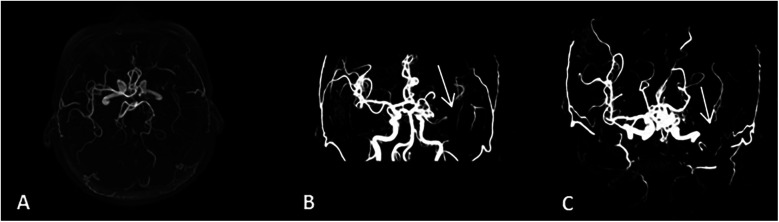
Head MRA results. Head MRA test shows no visualization of the left middle cerebral artery, indicating occlusion. **(A-C)** represent the basal view, anterior view, and superior view of the head MRA, respectively.

**Figure 2:**
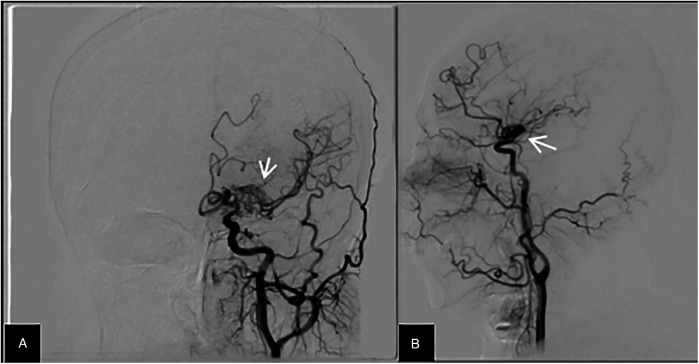
Head DSA results. Head DSA test reveals no main blood vessel in the M1 segment of the distal left middle cerebral artery, with multiple small arteries forming a network structure to supply blood. Imaging shows visualization of the M2 segment and distal vascular branches. (**A,B)** represent the anteroposterior (AP) and lateral views of the DSA, respectively.

A middle-aged female patient was admitted to our hospital for diagnosis and treatment due to intermittent dizziness lasting for two months. The patient had a history of hepatitis B for many years, a cesarean section over 20 years ago, and an appendectomy more than ten years ago. Neurological examination upon admission revealed no significant abnormalities. Laboratory tests showed no notable abnormalities in blood, urine, stool, occult blood test, coagulation profile, liver function, kidney function, or homocysteine levels. The lipid profile revealed a low-density lipoprotein (LDL) of 2.81 mmol/L. Cranial MRA indicated no visualization of the left middle cerebral artery (Figure 1). Color transcranial Doppler (TCD) showed reduced blood flow in the left middle cerebral artery, slower flow than the ipsilateral anterior and posterior cerebral arteries and the contralateral middle cerebral artery, and a relatively lower pulsatility index. Ultrasound findings suggested severe stenosis or occlusion of the left middle cerebral artery. Whole-brain angiography showed clear blood flow in the left common carotid artery and internal carotid artery; however, no main trunk of the distal left middle cerebral artery M1 segment was visualized, and a network of small arteries supplying blood was observed. Distal M2 segments and beyond showed visualization, and the left anterior cerebral artery was visible (Figure 2).

**Figure 3:**
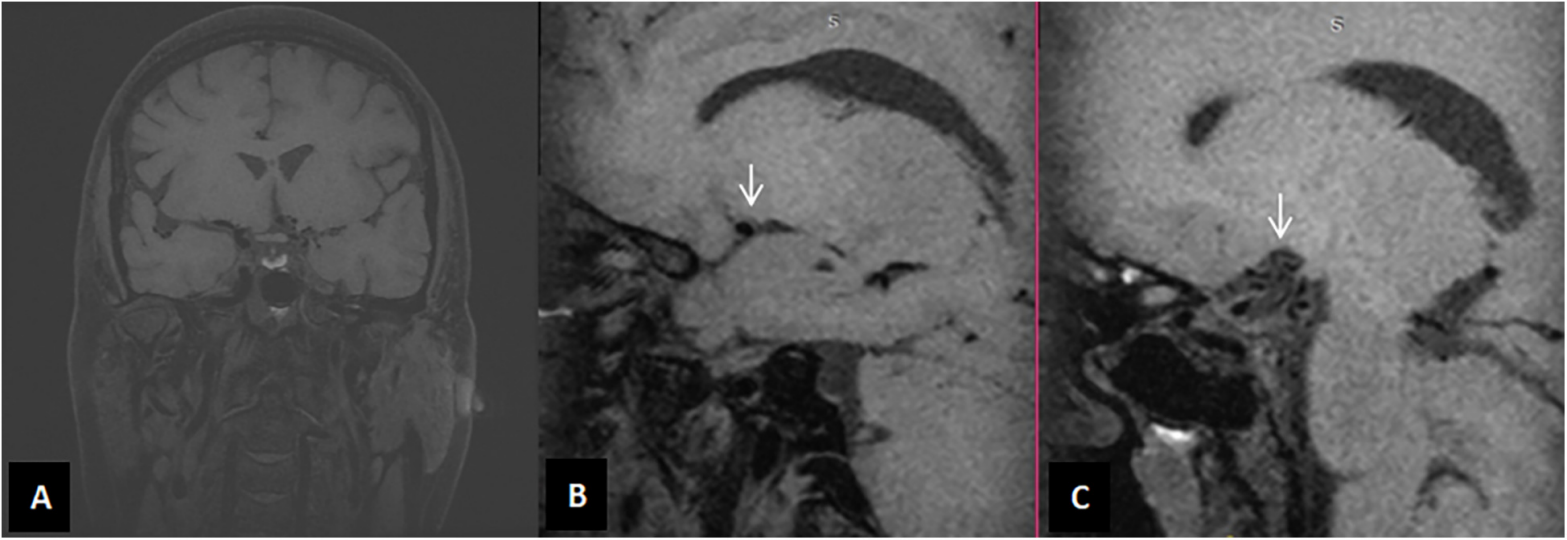
High-definition imaging of intracranial arterial plaques shows no visualization of the main trunk of LM1. Significant diameter reduction and vessel wall thickening are noted at the proximal end. Multiple tortuous black blood vessels are seen nearby, along with small vascular shadows connected to the cavernous sinus segment of the left internal carotid artery (LICA) and the left anterior cerebral artery (LA1). No thickening or narrowing of the intracranial arterial walls is observed, and there is no evidence of abnormal wall enhancement. **(A)** Coronal view of HRMRI, displaying the cerebral vasculature in the coronal plane. **(B)** Sagittal view of HRMRI, showing the right middle cerebral artery lumen (indicated by the white arrow). **(C)** Sagittal view of HRMRI, showing the location of the left middle cerebral artery (indicated by the white arrow).

High-resolution intracranial arterial plaque imaging revealed no visualization of the LM1 main trunk on plain scan or enhanced high-resolution black-blood sequence; the proximal segment showed significant narrowing with circumferential thickening of the vessel wall. The plain T1WI showed an isointense signal without significant enhancement, and multiple tortuous vascular lumens were observed nearby. Other arterial walls did not have thickening, stenosis, or wall enhancement. Changes at the origin of the LM1 matched the characteristics of T-MCA (Figure 3). The patient was ultimately diagnosed with left-sided T-MCA, with the consideration that her dizziness might be incidental.During hospitalization, the patient received intravenous infusions of Pentoxifylline, Salvia Miltiorrhiza Extract, and Xingnaojing Injection. Subsequently, her symptoms of dizziness were significantly alleviated, and she was discharged in improved condition. The patient was fully informed of the study's purpose, consented, and agreed to publish this case in a medical journal anonymously. The ethics committee of the research institution approved this study.

## Discussion

T-MCA, also known as embryonic unfused middle cerebral artery (MCA), is relatively rare in clinical practice, and there is currently no standardized nomenclature for it internationally (1–3). The middle cerebral artery (MCA) is ultimately formed through the regression and fusion of the retiform plexus originating from the distal part of the primitive anterior choroidal artery (Anterior Choroidal Artery, AChA) during the embryonic period. Failure in the regression and fusion of the retiform plexus can lead to variations of the MCA. The pathogenesis of T-MCA remains unclear, with two primary hypotheses currently under consideration. The mainstream hypothesis suggests that during embryonic development, the primitive middle cerebral artery is composed of multiple plexiform vascular networks, which normally fuse to form the typical middle cerebral artery. Failure of this fusion process results in the persistence of a plexiform arterial network in the M1 segment, presenting as a twig-like appearance (4, 5).Alternatively, T-MCA may represent an acquired condition. It is proposed that T-MCA could be a secondary consequence of chronic middle cerebral artery occlusion. This view is supported by the observation that unilateral M1 segment changes and the presence of normally developed distal MCA branches are less likely to occur embryologically, thus favoring an acquired etiology (6–8).

The clinical manifestations of T-MCA mainly include dizziness, headaches, strokes, and transient ischemic attacks. Studies have reported that some patients have unclear visualization of one side of the middle cerebral artery during cranial MRA as part of a routine examination, and DSA confirms the diagnosis of this condition. However, the patients themselves may be asymptomatic. In a review study by Onoue et al. (7, 9), 54% of patients reported headache symptoms, possibly related to hemodynamic disturbances associated with T-MCA. Although most T-MCA patients do not exhibit specific clinical symptoms, they can still lead to severe complications. Hemodynamic abnormalities in T-MCA patients can result in the rupture of T-MCA vessels or aneurysms, leading to intracranial hemorrhage, however, the natural course of this type of T-MCA is unknown. Some studies have reported that the probability of intracranial hemorrhage occurring in T-MCA patients ranges from 40% to 51% (1, 10). In addition, T-MCA leads to a state of hypoperfusion in the middle cerebral artery region, which can result in cerebral infarction or transient ischemic attacks. Therefore, early identification of T-MCA and implementation of appropriate interventions are crucial for treating patients.

The imaging examinations for T-MCA include DSA, MRA, and CTA, with DSA being the gold standard for diagnosis. Key diagnostic points for T-MCA on DSA include an abnormal tufted arterial network combined with stenosis or occlusion changes in the M1 segment and the presence of the Temporo-Limbic Network (TLN) with multiple channels connected to the *in situ* MCA (the distal M1 segment or proximal M2 segment). The distal branches of the MCA far from the TLN are close to average in diameter, with antegrade blood flow; the terminal internal carotid artery (ICA) and posterior circulation are unaffected; lenticulostriate arteries (LSA) originate from the TLN; and there are no meningeal collateral branches (11). The DSA findings of this patient are consistent with the imaging characteristics of T-MCA. High-resolution magnetic resonance imaging also has diagnostic value for T-MCA (12). After performing MRA on this patient, no visualization of the left middle cerebral artery M1 segment was found. Subsequent high-resolution magnetic resonance imaging indicated that the LM1 main trunk was not visible, with significant narrowing of the proximal segment, circumferential thickening of the vessel wall, and multiple tortuous black vascular lumens nearby. Some small vascular shadows were seen connected to the cavernous segment of the left internal carotid artery (LICA) and the left anterior cerebral artery (LA1). These findings suggest that the intracranial segment of the left internal carotid artery is connected to a network of vessels, which is diagnostically significant for T-MCA. Transcranial Doppler (TCD) and MRA have limited diagnostic value for this condition. Therefore, patients suspected of having T-MCA should be promptly evaluated with DSA in clinical practice.

T-MCA is primarily differentiated from moyamoya disease (MMD) in clinical practice (13). The differentiation is mainly based on the following aspects:(1) T-MCA shows only stenotic and obstructive changes in the M1 segment of the middle cerebral artery, replaced by a tufted vascular network (parallel to the course of the middle cerebral artery), while the distal vessels are relatively routine. In contrast, MMD exhibits not only stenotic and obstructive changes in the M1 segment but also a nearly complete disappearance of the distal vessels, with the tufted vessels oriented perpendicularly to the axis of the middle cerebral artery. (2) Blood supply in T-MCA can originate from the middle cerebral artery, internal carotid artery, anterior cerebral artery, and lenticulostriate arteries, with anastomoses converging in the Sylvian fissure at the M1 segment. In MMD, the middle cerebral artery region is primarily supplied by the middle cerebral artery, which is gradually replaced by tufted vessels originating from the cranial base around the basal ganglia. (3) T-MCA does not exhibit transdural collateral circulation, whereas transdural collateral circulation is joint in MMD. (4) Patients with T-MCA often have associated intracranial aneurysms, while patients with MMD have fewer instances of this complication. (5) The vascular structure in T-MCA is relatively stable, whereas MMD tends to progress over time. In this case, the patient presented with unilateral MCA involvement. DSA revealed a tufted vascular network in the M1 segment of the MCA, with patent distal vessels, thereby ruling out a diagnosis of MMD.

Currently, there are relatively few case reports on T-MCA. I have conducted a brief summary of the previously reported cases (14–19) (Table 1). From these six cases of T-MCA, it is evident that the clinical manifestations of T-MCA patients primarily include dizziness, headache, and neurological deficits. In cases with concomitant aneurysmal rupture, symptoms may be more severe and potentially life-threatening. Regarding imaging studies, MRA (Magnetic Resonance Angiography) can serve as an initial screening tool, while HRMRI (High-Resolution Magnetic Resonance Imaging) can provide certain diagnostic value. However, DSA (Digital Subtraction Angiography) remains the gold standard for the definitive diagnosis of T-MCA.There has yet to be a consensus on the treatment principles for T-MCA. Due to surgery's potential risks and complications, asymptomatic T-MCA patients may undergo conservative treatment first, with regular follow-up evaluations (1). For arterial aneurysms associated with tree—like middle cerebral artery (T-MCA), especially those that have ruptured, surgical treatment may be considered (15, 17).

**Table 1:** Summary of case reports related to T-MCA.

Case No.	Authors	Gender/Age	Symptoms	Imaging studies	Initial diagnosis	Final diagnosis	Aneurysm	Aneurysm location	Treatment
Case no. 1 (2016)	Uchiyama, et al.	Female/52 years	Sudden headache and transient weakness in the right hand	HRMRI, MRA, DSA	Severe stenosis of the left middle cerebral artery	Twig-like middle cerebral artery on the left side	No	–	Conservative treatment, no changes on follow-up
Case no. 2 (2017)	Lang, et al.	Female/54 years	Right-sided hemiplegia, global aphasia, severe headache, vomiting, and impaired consciousness	CT, DSA	Occlusion of the upper branch of the left middle cerebral artery and aneurysm of the middle cerebral artery	Twig-like middle cerebral artery involving the M2 segment on the left side	Yes	M2 segment of the left middle cerebral artery	Craniectomy and surgical clipping
Case no. 3 (2020)	Matsuo A, et al.	Female/17 years	In a lethargic state with mild dysarthria	MRA, DSA	Reticular and plexiform changes in the proximal right middle cerebral artery	Twig-like middle cerebral artery on the right side	No	–	Conservative treatment, no changes on follow-up
Case no. 4 (2021)	Takarada, Ayako et al.	Female/46 years	Sudden headache	CT, DSA	Bilateral absence or reticular appearance of the proximal middle cerebral artery	Same as initial	No	–	Bypass surgery
Case no. 5 (2024)	Kawakami M, et al.	Male/67 years	Sudden headache	CT, MRA, DSA	Reticular change in the proximal left middle cerebral artery	Twig-like middle cerebral artery on the left side	No	–	Conservative treatment, no changes on follow-up
Case no. 6 (2024)	Wang, Zhi-Gang et al.	Male/46 years	Intermittent dizziness	HRMRI, MRA, DSA	Anterior communicating artery aneurysm	Twig-like middle cerebral artery with anterior communicating artery aneurysm	Yes	Anterior communicating artery	Conservative treatment, no changes on follow-up

Asymptomatic T-MCA is challenging to identify clinically, making it essential to perform DSA or high-resolution MRI for suspected cases promptly. Regular follow-up is also necessary to confirm the final diagnosis in these patients.

## Data Availability

The original contributions presented in the study are included in the article/Supplementary Material, further inquiries can be directed to the corresponding author.

## References

[1] Seo B-S Lee Y-S Lee H-G Lee J-H Ryu K-Y Kang D-G. Clinical and radiological features of patients with aplastic or twiglike middle cerebral arteries. Neurosurgery. (2012) 70(6):1472–80. 10.1227/NEU.0b013e318246a510 22186843

[2] Liu H-M Lai D-M Tu Y-K Wang Y-H. Aneurysms in twig-like middle cerebral artery. Cerebrovascular Diseases. (2005) 20(1):1–5. 10.1159/000086119 15925875

[3] Cekirge HS Peynircioglu B Saatci I. Endovascular treatment of an “anterior cerebral artery” aneurysm in a patient with “embryonic unfused middle cerebral artery” anomaly: a case report. Neuroradiology. (2005) 47(9):690–4. 10.1007/s00234-005-1407-3 15940529

[4] Akkan K Ucar M Kilic K Celtikci E Ilgit E Onal B. Unfused or twig-like middle cerebral artery. Eur J Radiol. (2015) 84(10):2013–8. 10.1016/j.ejrad.2015.06.012 26123843

[5] Goto Y Oka H Hiraizumi S Okamoto T Nishii S Yamamoto H Aplastic or twig-like middle cerebral artery presenting with intracerebral hemorrhage during pregnancy: report of two cases. World Neurosurgery: X. (2019) 2:100018. 10.1016/j.wnsx.2019.100018 31218292 PMC6580884

[6] Takeda H Yanaka K Onuma K Nakamura K Ishii K Ishikawa E. Aplastic or twiglike middle cerebral artery with contralateral middle cerebral artery stenosis showing transient ischemic attack: illustrative case. J Neurosurg Case Lessons. (2022) 3(22):CASE22121. 10.3171/CASE22121 35734606 PMC9204927

[7] Soejima K Hiu T Shiozaki E Ogawa Y Ito T Honda K [Asymptomatic aplastic or twig-like middle cerebral artery associated with unruptured cerebral aneurysms at the origin (A1) of a collateral artery and the anterior communicating artery: a case report with multiple intracranial atherosclerotic stenoses]. Brain Nerve. (2021) 73(4):379–88. Japanese. 10.11477/mf.1416201771 33824225

[8] Ota T Komiyama M. Twig-like middle cerebral artery: embryological persistence or secondary consequences? Interv Neuroradiol. (2021) 27(4):584–7. 10.1177/15910199211024077 34096364 PMC8580538

[9] Inoue H Oomura M Nishikawa Y Mase M Matsukawa N. Aplastic or twig-like middle cerebral artery and cardiogenic cerebral embolism mimicking moyamoya disease with RNF213 polymorphism: a case report. Interv Neuroradiol. (2022) 28(6):634–8. 10.1177/15910199211062016 34913393 PMC9706272

[10] Matsunaga Y Izumo T Morofuji Y Horie N Hayashi K Matsuo T. Revascularization for aplastic or twiglike middle cerebral artery: a case report. J Stroke Cerebrovasc Dis. (2018) 27(5):e78–9. 10.1016/j.jstrokecerebrovasdis.2017.12.004 29310957

[11] Onoue K Nguyen TN Mian A Dasenbrock H Bedi H Abdalkader M. Twig-like middle cerebral arteries: clinical and radiological findings. Clin Imaging. (2021) 73:31–7. 10.1016/j.clinimag.2020.11.049 33296771

[12] Krishnamoorthy V Manley GT Jain S Sun S Foreman B Komisarow J Incidence and clinical impact of myocardial injury following traumatic brain injury: a pilot TRACK-TBI study. J Neurosurg Anesthesiol. (2022) 34(2):233–7. 10.1097/ANA.0000000000000772 33901061 PMC8536798

[13] Goto Y Nanto M Oka H Murakami N Nakagawa T Kimura S Radiological and clinical features of twig-like middle cerebral artery in comparison with moyamoya angiopathy: a multicenter retrospective study. J Neurosurg. (2022) 137:1–9. 10.3171/2022.2.JNS212338 35426829

[14] Uchiyama T Okamoto H Koguchi M Tajima Y Suzuyama K. [A case of aplastic or twig-like middle cerebral artery presenting with an intracranial hemorrhage two years after a transient ischemic attack]. No Shinkei Geka. (2016) 44(2):143–8. Japanese. 10.11477/mf.1436203246 26856268

[15] Lang M Moore NZ Witek AM Kshettry VR Bain MD. Microsurgical repair of ruptured aneurysms associated with moyamoya-pattern collateral vessels of the middle cerebral artery: a report of two cases. World Neurosurg. (2017) 105:1042.e5–1042.e10. 10.1016/j.wneu.2017.06.166 28698088

[16] Matsuo A Hiu T Ito T Moritsuka T Honda K Kawahara I [Aplastic or twig-like middle cerebral artery with cortical subarachnoid hemorrhage and reversible cerebral vasoconstriction syndrome during the postpartum period in a juvenile female: a case report]. No Shinkei Geka. (2020) 48(5):435–44. Japanese. 10.11477/mf.1436204206 32434955

[17] Takarada A Yanaka K Onuma K Nakamura K Takahashi N Ishikawa E. Aplastic or twig-like middle cerebral artery harboring unruptured cerebral aneurysms treated by clipping and bypass surgery: illustrative case. J Neurosurg Case Lessons. (2021) 2(9):CASE21360. 10.3171/CASE21360 35854945 PMC9265206

[18] Kawakami M Murai S Kusaka N Baba F Inoue Y Miyake H Non-aneurysmal subarachnoid hemorrhage in aplastic or twig-like middle cerebral artery: a case report and literature review. J Stroke Cerebrovasc Dis. (2024) 33(3):107582. 10.1016/j.jstrokecerebrovasdis.2024.107582 38237811

[19] Wang Z-G Chen L-A Liu Q Wang B. Bilateral twig-like middle cerebral artery: a case report. Asian J Surg. (2024) 47(4):2002–3. 10.1016/j.asjsur.2023.12.203 38212220

